# Evaluation of selected biological capacities of *Baeckea frutescens*

**DOI:** 10.1186/s12906-015-0712-6

**Published:** 2015-06-17

**Authors:** Suerialoasan Navanesan, Norhanom Abdul Wahab, Sugumaran Manickam, Kae Shin Sim

**Affiliations:** Institute of Biological Sciences, Faculty of Science, University of Malaya, 50603 Kuala Lumpur, Malaysia; Biology Division, Centre for Foundation Studies in Science, University of Malaya, 50603 Kuala Lumpur, Malaysia; Rimba Ilmu Botanic Garden, Institute of Biological Sciences, Faculty of Science, University of Malaya, 50603 Kuala Lumpur, Malaysia

**Keywords:** *Baeckea frutescens*, Antioxidant, Lung carcinoma, Acute oral toxicity

## Abstract

**Background:**

*Baeckea frutescens* is a natural remedy recorded to be used in curing various health conditions. In Peninsular Malaysia, *B. frutescens* is found on the mountain tops, quartz ridge and sandy coasts. To our knowledge, there is only limited published literature on *B. frutescens*.

**Methods:**

*B. frutescens* leaf crude methanol and its fractionated extracts (hexane, ethyl acetate and water) were prepared. Folin-Ciocalteau’s method was used for the measurement of total phenolic content of the extracts. The antioxidant activity was measured by the scavenging activity on DPPH (1,1-diphenyl-2-picrylhydrazyl) radicals, reducing power assay through the Prussian blue complex formation, the metal chelating assay as well as the β-Carotene-linoleic acid system assay. The cytotoxic activity of the extracts were evaluated against two lung carcinoma cell lines with varying molecular characteristics using the MTT [3-(4, 5-dimethylthiazol-2-yl)-2, 5-diphenyltetrazolium bromide] assay. Lastly the toxicity of the crude methanol extract was evaluated using the acute oral toxicity experiment.

**Results:**

The methanolic extract with highest phenolic content showed the strongest *β*-carotene bleaching inhibition, whilst the water extract exhibited the highest activity in metal chelating and reducing power assays. The hexane extract displayed a mild cytotoxic effect on both A549 and NCI-H1299 human lung carcinoma cell lines. No mortalities and no adverse effects were observed in the acute oral toxicity investigation at the highest dose of 5000 mg/kg.

**Conclusion:**

The findings in the present study suggest *B. frutescens* may be considered as a safe source of compounds with antioxidant and cytotoxic properties for therapeutic and functional food applications.

## Background

Alternative therapy has been extensively used throughout time in the prevention and treatment of various diseases. Over the past twenty years, traditional medicine has been gaining widespread attention among the general public as well as researchers due to the fact that they are natural and readily accessible [1]. The medicinal plants not only provide a wide range of vitamins and nutrients to the human diet, but also possess chemical compounds which might have the potential to be used as therapeutic agents. It can be said that natural products do play a crucial role in development of drugs which will lead towards the treatment and prevention of various illnesses [2].

*Baeckea frutescens*, which is found in Peninsular Malaysia, Sumatra and the coastal areas of southern China and Australia, is claimed to possess anti-bacterial, anti-dysentery, anti-pyretic and diuretic activities [3]. In Peninsular Malaysia, *B. frutescens* is found on mountain tops, quartz ridge and sandy coasts of the eastern parts [4, 5]. The leaves release an aromatic fragrance when crushed; the flowers are minute, solitary or in pairs with white petals [4]. *B. frutescens* is believed to be effective in treating influenza, coryza, epistaxis, sunstroke, fever, headache, measles, colic, abdominal pain, dyspepsia, jaundice, haemorrhagic dysentery and irregular menstrual cycles. In Malaysia and Indonesia, *B. frutescens* is one of the ingredients utilized in traditional medicines during confinement and used in massaging postpartum women for the treatment of body aches and numbness of the limbs. The essential oil extracted from this plant is used in the treatment of rheumatism by the locals.

To our knowledge, there is only limited published literature on *B. frutescens*. An investigation by Fujimoto et al. [6] reported that one of the ethanolic leaf extract isolates of *B. frutescens* exhibited strong cytotoxic activity against L1210 leukaemia cells. The phytochemical study conducted by Makino and Fujimoto [7] reported the three flavanones isolated from *B. frutescens* leaf extracts also showed promising cytotoxicity against L1210 leukaemia cells. A later report by Hwang et al. [8] indicated anti-cariogenic, anti-malarial and anti-babesial activities of *B. frutescens*.

The current study was aimed to investigate the total phenolic content, antioxidant potential, cytotoxic activity and acute oral toxicity of *B. frutescens* leaf extracts. The outcome derived from this research will provide a strong foundation for further in depth studies to be performed using *B. frustescens*.

## Methods

### Plant sample collection and identification

The fresh leaves of *B. frutescens* were collected from Klang Gates, Ampang Jaya, Kuala Lumpur, Malaysia on April 2012. The plants were identified by Dr Sugumaran Manickam, of Institute of Biological Sciences, Faculty of Science, University of Malaya, Malaysia. A voucher specimen (KLU 47909) was deposited at the herbarium of the Rimba llmu Botanic Garden, Institute of Biological Sciences, Faculty of Science, University of Malaya, Kuala Lumpur, Malaysia.

### Extraction and fractionation

Extraction was done using the solvent extraction method, whereby fresh *B. frutescens* leaves (17.20 kg) were washed, oven dried (40 to 45 °C) and ground to fine powder (10.00 kg). The ground leaves (500 g) were extracted with methanol (3x 1 litre) at room temperature, yielding a dark green crude methanol extract (230.10 g, 46.02 %) after evaporating the excess solvent with a rotary evaporator. Upon setting aside 10.00 g of the methanol extract for further testing, the balance 220.10 g of methanol extract was further fractioned using hexane to give a hexane-soluble extract (17.50 g, 7.95 %) and hexane-insoluble residue. The hexane-insoluble residue was then partitioned between ethyl acetate and water (1:1, 100 ml: 100 ml) to give an ethyl acetate-soluble extract (16.80 g, 7.63 %). The water layer was freeze-dried to give a brown water extract (12.90 g, 5.86 %). All the extracts were stored at 4 °C in the dark until further analysis.

### Determination of total phenolic content

The concentrations of phenolic compounds in the extract and fractions of *B. frutescens* were measured according to a modified Folin-Ciocalteu method which utilizes the 96-well plates as described by Teoh et al. [9]. Firstly, 25 μl Folin-Ciocalteu reagents were added to 10 μl of the solubilized extract and its fractions (ranging from 0 to 0.5 mg/ml) in the wells of a 96-well plate. This was followed by incubation for 5 min at room temperature (27 ± 1 °C), after which 25 μl of 20 % (w/v) sodium carbonate was added to the mixture followed by distilled water to a final volume of 200 μL per well. Following this, the plate was incubated for 30 min at room temperature (27 ± 1 °C) and the absorbance was read at 760 nm against a blank (distilled water) using a Multiskan GO micro plate reader (Thermo Fisher Scientific). Triplicates were performed for each sample to ensure reproducibility in the results obtained. Galic acid (0–1000 mg/L) was used to plot a standard curve. The results were expressed as milligram of Gallic acid equivalents per gram of sample (mg of GAE/g of sample) using the gallic acid calibration plot with the following formula:

$$ \mathrm{Total}\ \mathrm{phenolic}\ \mathrm{content} = \left(\ \mathrm{y} + 0.0104\right)/0.002,\ {\mathrm{R}}^2 = 0.9855 $$

y is the absorbance value of extracts after subtraction of control.

### DPPH radical scavenging activity

The free radical-scavenging potential of the extracts and its fractions were analysed according to previously reported method of Teoh et al. [9] which utilizes the 96-well plates. Briefly, an aliquot (50 μl) of extract at various concentration (ranging from 0 to 0.5 mg/ml) was mixed with 150 μl of a DPPH solution in ethanol (0.3 mM) into each well. Instead of the extract, 50 μl of distilled water was used for the blank. The variation in absorbance upon incubation at room temperature was measured at 515 nm using a Multiskan GO micro plate reader (Thermo Fisher Scientific). All assays were performed in triplicates to ensure their reproducibility. A standard curve was obtained using different concentrations (0–0.5 mg/ml) of BHA which was used as positive reference standard solution. The scavenging activity (%) on DPPH radical was calculated according to the following equation:

$$ \mathrm{Scavenging}\ \mathrm{activity}\ \left(\%\right) = \left[\left(\mathrm{A}\ \mathrm{control}\hbox{-} \mathrm{A}\ \mathrm{sample}\right)\ /\mathrm{A}\ \mathrm{control}\right]\kern0.5em \times 100\% $$

A control is the absorbance of the control and A sample is the absorbance of the tested extract. The scavenging ability of the extracts was expressed as EC_50_ value, which is the effective concentration at which 50 % of DPPH radicals were scavenged. The EC_50_ values were obtained from the graph of scavenging activity (%) versus concentrations of samples. BHA was used as a positive reference standard.

### Reducing power assay

The reducing power of *B. frutescens* extract and fractions was determined by the formation of Prussia blue complex in the samples according to the modified method of Zubia et al. [10]. Briefly, 200 μl of the extracts at various concentrations were mixed with 200 μl phosphate buffer (0.2 M, pH 6.6) and 200 μl potassium ferricyanide [K_3_Fe(CN)_6_] (1 %). The mixture was left to cool down after incubation for 30 min at 50 °C before adding 200 μl of trichloroacetic acid (10 %). 125 μl of the solution was then transferred to a 96-well plate before finally adding 20 μl of 0.1 % FeCl_3_ . 6H_2_O . Changes in the absorbance caused by the reaction were quantified using a Multiskan GO micro plate reader (Thermo Fisher Scientific) by reading the absorbance at 620 nm. The absorbance data was then expressed as a percentage of inhibition by comparison to a negative control (blank). The assay was carried out in triplicate for each sample and also the positive controls (BHA).

### Metal chelating assay

The chelating activity of *B. frutescens* extract and fractions was experimented according to the method of Teoh et al. [9]. 50 μL of the extracts with varying concentrations was mixed with 5 μL of ferrous chloride hexahydrate (2 mmol/l) and 130 μl of deionized water before incubation for 5 min. Following this, the reaction was initiated by adding 15 μL of ferrozine (5 mmol/l) to each well and was then left aside for around 10 min to reach equilibrium. The absorbance was then read 562 nm using a Multiskan GO micro plate reader (Thermo Scientific). EDTA was used as positive reference standard for the experiment. The metal chelating percentage was calculated as follows:

$$ \%\ \mathrm{of}\ \mathrm{metal}\ \mathrm{chelating}=\left[\left(\mathrm{Acontrol}\hbox{-} \mathrm{Asample}\right)/\mathrm{Acontrol}\right]\times 100\ \%. $$

**A control** is the absorbance of the negative control and **A sample** is the absorbance of the tested extract. All the experiments were carried out in triplicates to ensure their reproducibility.

### *β*-Carotene-linoleic acid system assay

The modified method of Murugan and Iyer [11] was used to test the bleaching ability of *B. frutescens* extracts against *β*-carotene in the current study Firstly, 5 mg of β-carotene was dissolved in 50 ml of chloroform and 1 ml of this solution was mixed with 200 μl of linoleic acid and 600 mg of Tween-20. Removal of chloroform from the mixture was done through rotary evaporation at 40 °C under vacuum before slowly adding 50 ml of distilled water oxygenated by air-bubbling. While adding the oxygenated distilled water, the semi-solid was subjected to vigorous stirring to form an emulsion, of which 200 μl will then be added to each well that has been preloaded with 10 μl of the samples or the positive control (BHA). Methanol was used as a blank for this experimentation. The absorbance was immediately read at 470 nm using a Multiskan GO micro plate reader (Thermo Fisher Scientific) upon addition of the emulsion (t = 0). The plate was then covered and incubated at 50 °C for two hours. The change in absorbance was monitored by reading the plate every 20 min throughout the incubation period. The samples antioxidant activities were expressed as the percentage of β-carotene bleaching inhibition. All assays were performed in triplicates. The following formula was used for that purpose:

$$ \mathrm{Antioxidant}\ \mathrm{activity}\ \left(\%\right) = \left[1\hbox{-}\ \left(\mathrm{A}{\mathrm{e}}_0\hbox{-}\ \mathrm{A}{\mathrm{e}}_{\mathrm{t}}\right)\ /\ \left(\mathrm{A}{\mathrm{c}}_0\hbox{-}\ \mathrm{A}{\mathrm{c}}_{\mathrm{t}}\right)\right] \times 100\% $$

Ae_0_ is the absorbance of sample at t = 0 and Ae_t_ is the absorbance of the sample at t = 2 h. Ac_0_ is the absorbance of control at t = 0 and Ac_t_ is the absorbance of the control at t = 2 h.

### MTT Cytotoxicity assay

Two human lung cancer cell lines, A549 and NCI-H1299 were purchased from American Type Culture Collection (ATCC, USA). Both A549 and NCI-H1299 cells were cultured and maintained in RPMI 1640 medium supplemented with 10 % foetal bovine serum, 2 % penicillin or streptomycin and 1 % Amphotericin B. The cells were cultured in a 5 % CO_2_ incubator (Shel Lab water-jacketed) kept at 37 °C in a humidified atmosphere. The MTT cytotoxicity assay was carried out using similar methods to those previously described by Mosmann [12]. Briefly, cells were plated at a density of 7000 cells per well into a 96-well plate and left to incubate overnight. The cells were then treated with *B. frutescens* extracts that were initially diluted to intended concentration (ranging from 100 to 5 μg/ml). The diluted extracts only contained 0.5 % DMSO (dimethyl sulfoxide) which was found to be non-toxic towards the cell lines used (data not shown). The plates were left to incubate for 72 h. The 20 μl of the MTT reagent (0.5 mg/ml) was then added followed by three hour incubation period before removing the media and MTT reagent and finally dissolving the formazan crystals with 200 μl of DMSO. The absorbance was read using a Multiskan GO micro plate reader (Thermo Fisher Scientific) at 570 nm with 650 nm as a reference wavelength. Cisplatin was used as positive reference standard. All assays were performed in triplicates.

### Acute oral toxicity

The current acute oral toxicity study was performed on twelve healthy male Sprague–Dawley rats (aged 8–12 weeks, weighing 200–250 g) which were obtained from Laboratory Animal Centre, Faculty of Medicine, University of Malaya, Malaysia. The experiment protocol was approved by the Institutional Animal Care and Use Committee, University of Malaya (UM IACUC) with ethical number: ISB/29/06/2012/SKS (R) before the acute oral toxicity assay was carried out. The rats were placed in stainless steel cages in a ventilated room with 12-h light/dark cycle at room temperature (approximately 25 °C) with constant humidity and were allowed to acclimatize to the room condition for a week before experimentation. The rats were given free access to water and food except for 12 h prior to dosing.

The toxicity study of *B. frutescens* extracts was performed according to the procedure described by Teoh et al. [9] referring to Organization for Economic Cooperation and Development (OECD) guideline 423 [13]. The rats were divided into four groups (three treatment groups and one control group). Each treatment group was dosed with 300, 2000 and 5000 mg of the extract per kilogram of body weight respectively. The control group was just treated with the vehicle, which was 0.3 % carboxymethyl cellulose suspension. Following the 12 h of fasting period, the rats were weighed and the dosage was calculated using the body weight as a reference and the volume of the extracts given to the rats were 1 ml/100 g of the body weight. A syringe attached to a stainless steel ball-tipped gavage needle was used to orally administer the dosage to the rats. Food was provided to the rats four hours after the treatment. The rats were weighed and observed for signs of toxicity effects (mortality, changes in behaviour and physical appearances) within the first four hours after treatment and daily for a further period of 14 days. The experiment was repeated twice to ascertain the reproducibility of results.

### Statistical analysis

All results in determination of total phenolic content, antioxidant assays and MTT cytotoxicity assay were obtained in triplicates and data were expressed as mean ± SD. Data for the antioxidant assays were subjected to analysis of variance (ANOVA) and means were separated by Duncan multiple range test at P < 0.05 significant level. IC_50_ values for the MTT cytotoxicity assays were obtained using the GraphPad Prism software.

## Results and Discussion

### Total phenolic content of *B. frutescens* extracts

Phenolic compounds refer to plant substances which possess one or more aromatic ring that bears one or more hydroxyl substituents and, this characteristic gives phenolic compounds the potential quench free radicals, thus making them good antioxidants. There was a wide range of phenolic concentrations present in the methanolic crude extract of *B. frutescens* as well as in its fractionated extracts (hexane, ethyl acetate and water), as shown in Table 1.

**Table 1:** The total phenolic content of *B. frutescens* extracts

Extract	Concentration of total phenolic (mg of GAEs/g of extract)
Methanol	27.90 ± 1.15^d^
Hexane	15.32 ± 1.82^b^
Ethyl acetate	22.15 ± 1.46^c^
Water	4.17 ± 2.08^a^
BHA*	44.06 ± 4.61^e^

*Positive reference standard; GAEs, Gallic acid equivalents; Values are expressed as mean ± standard deviation (*n* = 15), in each column different letters (a–e) means significantly different (p < 0.05, ANOVA)

Among the extracts, the methanol extract exhibited a significantly higher phenolic content (p < 0.05), followed by the ethyl acetate extract and the hexane extract. BHA, which was used as a positive control showed the significantly highest phenolic content among all the tested samples (p < 0.05). The water extract presented the lowest phenolic content among all the tested extracts. The results indicated that most of the phenols were present in the crude methanol extract and least in the water extract of *B. frutescens*. According to Murugan and Iyer [14], molecules such as flavonoids and phenols are generally more soluble in methanol, chloroform and ethyl acetate. The current result could also be attributed to the fractionation process where different phenols present in methanol extract were then separated into different solvents based on their solubility.

### Antioxidant capacity of *B. frutescens* extracts

Most antioxidant assays are only able to measure a single mechanism or pathway as opposed to the complexity of interactions between antioxidants in vivo. In order to bypass this shortcoming, four simple, fast and reliable biochemical assays i.e., the DPPH radical scavenging assay, the reducing power assay, the metal chelating assay and the *β*-carotene-linoleic acid system assay, were used to assess the antioxidant capacity of *B. frutescens* extracts.

The ability of extracts to scavenge free radicals is related to hydrogen atoms or electron donation capabilities and the conformations of the antioxidant compounds [15]. In the present study, DPPH radical scavenging capacity was used to study the radical scavenging effects of the extracts as DPPH is a stable free radical that has a characteristic absorption at 517 nm which can be conveniently measured by a spectrophotometer. The data obtained in current DPPH assay was defined as the concentration of extracts needed to effectively decrease the initial concentration of the DPPH radicals by 50 % (EC_50_). The lower the EC_50_ values presented, the higher the radical scavenging ability of the extract. The blank control was used as a reference point to determine the effectiveness of the samples in scavenging the radicals by electron transfer.

The DPPH radical scavenging activities of *B. frutescens* extracts were shown in Table 2, as comparable with BHA which is a known antioxidant. The scavenging effects of *B. frutescens* extracts on DPPH radicals were in the following order: ethyl acetate > methanol > water > hexane. The ethyl acetate presented a significantly higher antioxidant activity (p < 0.05) at 0.084 mg/ml; the methanol and water extracts exhibited similar amount of scavenging activity with the EC_50_ values of 0.101 and 0.110 mg/ml, respectively. The hexane extract presented the weakest ability to scavenge DPPH free radicals with the EC_50_ value of 0.347 mg/ml. As mentioned earlier, molecules such as flavonoids and phenols are generally more soluble in methanol, chloroform and ethyl acetate. Taking this point together with the generally accepted statement that the total phenolic content usually correlates with other electron transfer based assay, we are able to derive a possible explanation as to the relatively good scavenging activity exhibited by the ethyl acetate fraction and methanol extract of the plant.

**Table 2:** The antioxidant capacity of extracts in DPPH radical scavenging, reducing power and metal chelating assay

Extract	EC_50_ values (mg/ml)		
	DPPH radical scavenging activity	Reducing power Assay	Metal chelating Assay
Methanol	0.101 ± 0.001^c^	0.041 ± 0.004^b^	0.067 ± 0.011^c^
Hexane	0.347 ± 0.005^e^	0.162 ± 0.002^d^	0.047 ± 0.011^b^
Ethyl acetate	0.084 ± 0.001^b^	0.033 ± 0.001^a^	0.158 ± 0.004^d^
Water	0.110 ± 0.001^d^	0.026 ± 0.002^a^	0.039 ± 0.005^a^
BHA*	0.030 ± 0.001^a^	0.051 ± 0.001^c^	-
EDTA*	-	-	0.042 ± 0.001^a^

*Positive reference standard; Values are expressed in μg/ml as mean ± standard deviation (*n* = 3), in each column different letters (a–d) means significantly different (p < 0.05, ANOVA)

The reducing power assay together with DPPH free radical scavenging assay are common biochemical assays used to assess the direct involvement of extracts in enhancing the primary antioxidant activity. In the reducing power assay, the reducing power capabilities of *B. frutescens* extracts were determined through its ability of direct electron donation in the reduction of ferric cyanide to ferrous cyanide. The higher the level of reductive capability that is possessed by the tested extract, the higher the absorbance value due to a larger number of Prussian blue colour complexes formed. The absorbance value was converted to percentage of reducing capabilities and the EC_50_ value was derived to obtain a better comparison between the tested extracts. The EC_50_ value was defined as concentration of extracts needed to effectively reduce ferric cyanide to ferrous cyanide by 50 %. The lower the EC_50_ values presented, the higher the reducing power of the extract.

The result in the reducing power assay was consistent with those obtained in the DPPH radical scavenging assay. The water, ethyl acetate and methanol extracts produced reasonable good antioxidant capabilities while the hexane extract exhibited the lowest reducing power (Table 2). It is interesting to note that all the extracts, with the exception of hexane extract, exhibited a significantly stronger reducing capability (p < 0.05) when compared to the positive reference standard (BHA). Since both the DPPH scavenging assay and reducing power assay are based on the electron transfer principles, it is not surprising that similar trends were observed in both studies. The presence of phenolic compounds in methanol and the ethyl acetate extracts could be responsible for their radical scavenging and reducing capabilities. As for the water fraction, it most likely contains other water soluble antioxidant (Vitamin C, aromatic amines, Cu(I), Fe (II) and ascorbic acid to name a few examples).

As antioxidant activities may be attributed to more than one mechanism, the ability of metal ion chelation of *B. frutescens* extracts was also tested in the current study. Metal ion chelation has an indirect antioxidant effect as oxidative damages may be promoted by certain transition metals such as iron and copper, and such oxidative reaction which occurs in vivo is involved in development of certain neurodegenerative diseases [16, 17]. The metal chelating assay quantifies the capability of the test samples in binding to oxidation promoting ferrous ion. Successful chelating of the ferrous ion will lead to the disruption in the formation of Fe(II) − ferrozine complex. This reaction will determine the ability of the extract to function as a secondary antioxidant through the prevention in forming of free radicals. As shown in Table 2, the water extract of *B. frutescens* exhibited a statistically significant higher metal chelating activity with an EC_50_ value of 0.039 mg/ml compared to the other extracts. This was marginally better than EDTA which was the positive reference standard (EC_50_ value of 0.042 mg/ml). On the other hand, ethyl acetate showed the weakest metal chelating ability with an EC_50_ value of 0.158 mg/ml. The metal chelating activity of all extracts differed significantly (p < 0.05) from each other. The strong ion chelating capacity of water extract could be explained based on previous reports which suggested water-soluble compounds such as polysaccharides do have capabilities to chelate metal ions in an approach similar to phenols [18]. The previous study by Wang et al. [18] also reported that polyphenols are not effective cheaters of transition metals when compared with polysaccharides, proteins or peptides which might play a more important role in the chelating effects. This statement is consistent with the current result obtained from the Folin–Ciocalteu assay (Table 1) and metal chelating assay (Table 2) which showed that the water extract with the lowest phenolic content exhibiting the strongest metal chelating ability while the methanol and ethyl acetate extracts exhibited a higher phenolic content coupled with a low metal chelating activity.

The antioxidant capabilities of *B. frutescens* extracts were also assessed using the *β*-carotene-linoleic acid system which involves linoleic acid that plays a role in generating radicals through spontaneous oxidation promoted by thermal induction. The test extract added to *β*-carotene-linoleic acid system was expected to neutralise the radicals by inhibiting the discolouration of *β*-carotene which can be quantified by measuring the absorbance. A high absorbance value can be translated to a high antioxidant capacity. The assay was done in the presence of increasing concentrations of extracts under evaluation across a fixed period of time.

As shown in Table 3, all *B. frutescens* extracts showed progressive increment in *β*-carotene bleaching inhibition with increasing concentrations, thus suggesting the presence of antioxidant compounds in the extracts. Generally, the methanol extract showed a significantly (p < 0.05) higher antioxidant activity at all the tested concentrations compared to the other extracts with percentage of inhibition ranging from 83.35 % to 87.38 %. In fact, the methanol extract produced a better β-carotene bleaching inhibition activity compared to BHA which was the positive reference standard, with the exception of the highest tested concentration (0.50 mg/ml), where it was lower by just under two percentage. At the highest tested concentration of extracts (0.50 mg/ml), the methanol extract exhibited the highest bleaching inhibition among the extracts, followed by hexane, ethyl acetate and water. A similar trend was observed in the total phenolic content (Table 1), suggesting possibility of a co-relationship between the amount of phenolic compounds present and the *β*-carotene bleaching inhibition of the extract.

**Table 3:** *β*-Carotene bleaching inhibition of *B. frutescens* extracts

Concentration (mg/ml)	*β*-Carotene bleaching inhibition (%)				
	0.01	0.05	0.10	0.25	0.50
Methanol	83.35 ± 3.20^aq^	85.63 ± 0.10^aq^	85.31 ± 0.30^aq^	86.70 ± 0.70^ap^	87.38 ± 0. 50^ap^
Hexane	78.43 ± 0.10^bt^	79.99 ± 0.20^bs^	80.92 ± 0.10^br^	81.86 ± 0.50^bq^	84.10 ± 0.80^bp^
Ethyl Acetate	66.03 ± 2.50^cs^	70.14 ± 0.20^cr^	71.92 ± 0.30^dr^	74.75 ± 0.70^dq^	78.80 ± 0.20^cp^
Water	66.98 ± 0.20^cs^	70.96 ± 0.80^cr^	73.18 ± 0.20^cq^	74.38 ± 0.10^dp^	74.60 ± 0.50^dp^
BHA*	62.18 ± 1.30^ds^	65.21 ± 1.00^ds^	69.52 ± 1.00^er^	78.62 ± 1.00^cq^	89.61 ± 4.20^ap^

*Positive reference standard; Values expressed are mean ± standard deviation (*n* = 3). For different extracts with the same concentration, means in the same column with different letters (a–e) were significantly different (p < 0.05, ANOVA). For the same extract or standard with different concentrations, means in the same row with different letters (p–t) were significantly different (p <0.05, ANOVA)

A dissimilar pattern was observed when comparing the β-carotene – linoleic acid system with the pervious antioxidant assays (DPPH radical scavenging assay, reducing power assay and metal chelating assay). This indicates that when using different methods, the same antioxidants may yield significantly different activity depending upon various mechanisms of antioxidant action [19, 20]. Generally, antioxidant activity depends on the composition of the extracts and the assay methods [20].

### MTT Cytotoxicity assay

The MTT assay was conducted in order to assess the cytotoxic activity of *B. frutescens* extracts on two human lung cancer cell lines, which were A549 and NCI-H1299. The A549 cell line has a functional wild type *p*53 expression while NCI-H1299 cell line is *p*53 deficient [21]. By means of using these two cell lines, the role of the extracts in causing cell death through the *p*53 gene can be assessed. The current study focused on lung cancer cell lines as *B. frutescens* has not been tested with lung cancer cells. Previous study by Fuijmoto et al. [6] as well as Makino and Fujimoto [7] reported that *B. frutescens* extract exhibited strong cytotoxic effect against L1210 leukaemia cells. According to the US National Cancer Institute (NCI) plant screening program, plant extracts are considered cytotoxic should the IC_50_ value be 20 μg/ml or less, following incubation between 48 to 72 h [22].

As shown in Table 4, all *B. frutescens* extracts showed IC_50_ values more than 20 μg/ml and did not managed to exert a cytotoxic effect against the tested cancer cell lines according to the criteria set by the US NCI plant screening program. Among all extracts, the hexane extract displayed the strongest cytotoxic activity with an IC_50_ of 56.24 and 26.70 μg/ml against A549 and NCI-H1299 cancer cells, respectively. The hexane extract seems to have a greater effect against the H1299 (p53 deficient) compared to the A549 cells (functional p53 gene). This could be an indication that the tumour suppressor gene has no or minimal involvement in the mechanism of cell death induced by the hexane extract.

**Table 4:** Cytotoxic activity (IC_50_ values) of *B. fructesans* extracts against A549 and NCI-H1299 cell lines

Extract	Cytotoxicity (IC_50_) in μg/ml	
	A549	NCI-H1299
Methanol	>100	93.20 ± 2.60
Hexane	56.24 ± 0.03	26.70 ± 0.23
Ethyl Acetate	>100	>100
Water	>100	>100
Cisplatin*	8.70 ± 1.30	21.41 ± 3.99

*Positive reference standard; Values are expressed in μg/ml as mean ± standard deviation (*n* = 3)

The lack of ability to disrupt the biochemical pathways present in the cancer cells may have been a cause as to why the extracts from this plant failed to exert a substantial cytotoxicity effect against the tested cancer cells. Moreover, two or more component present in the extracts could either be working synergistically together or antagonistically against each other which may be the reason for the lack of acceptable level cytotoxicity. This can be associated with the concept of synergism and antagonism between the compounds present in the extracts. It is not uncommon for crude extracts to possess a lower cytotoxic activity as compared to its isolates. As such the components should be isolated and re-evaluated.

### Acute oral toxicity of *B. frutescens* methanol extract

Toxicity is a state of adverse effects due to interactions between toxicants from poisonous substance with the living cells [23]. Evaluating the toxic potential of particular substance is crucial as exposure to toxicant may lead to adverse or hazardous effects on human beings. Thus, it is highly important to ensure the substance is safe before exposing it to humans through consumption. In the current study, an acute oral toxicity testing was conducted on Sprague–Dawley rats in order to determine the toxicity of *B. frutescens* extract. This was done to evaluate the safety of using this plant extract for pharmacological studies which will lead towards usage in humans. No toxic symptoms were observed on the test subjects throughout the whole experimentation which lasted for 14 days. Both extract-treated group and vehicle-treated group did not exhibit any abnormalities in behaviour, breathing, disruption in food and water consumption, skin effects and hair loss. A much more important outcome is that none of the tested rats encountered mortality throughout the 14 day toxicity study.

**Fig. 1:**
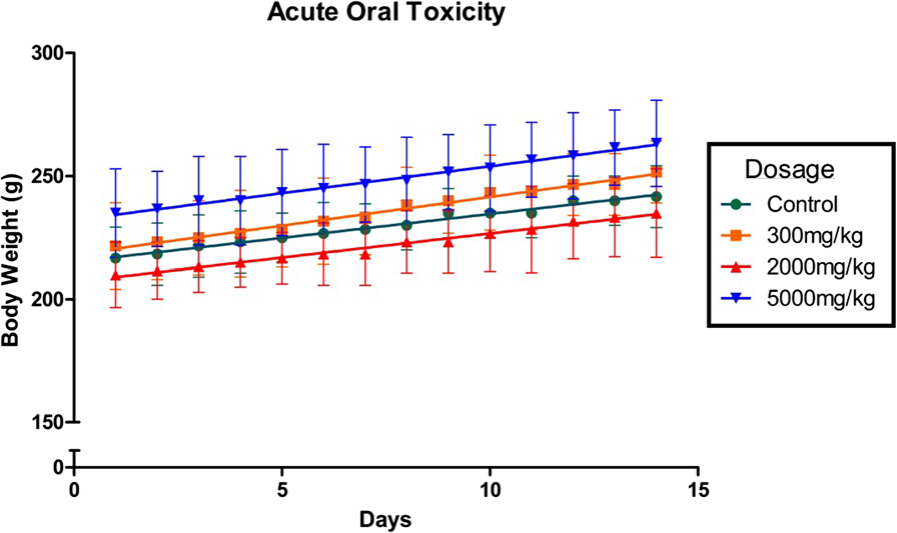
The effect of *B. frutescens* methanol extract on rat body weight

All the rats showed no sign of distress or displayed any symptoms associated with toxicity. The increase in body weight of the rats gradually with time indicates normal growth (Fig. 1). This is a clear indication that the extract tested has no significant amount of toxicity in reference with the growth of the rats. This factor combined with the observation that there were no disturbances in food and water intake could mean that there were no disruptions the animal’s metabolism [23].

Thus, it can be concluded that the methanolic extract of *B. frutescens* is non-toxic towards the rats. This study also indicates the Lethal Dose LD_50_ value for this extract is more than 5000 mg/kg. In accordance with OECD guideline for testing of chemicals, the methanolic extract of *B. frutescens* falls in category 5, where the LD_50_ exceeds 5000 mg/kg [13]. This is the lowest class of toxicity outlined by OECD. Any substance with a LD_50_ which exceeds 5000 mg/kg, which was administered orally to the animal, is considered as safe and non-toxic [24].

## Conclusion

The beneficial properties of natural products are endless. Modern science is just starting to appreciate nature and its large repository of naturally occurring biochemical compounds. There is an apparent shift from synthetic chemicals to natural chemicals in various fields, be it in food chemistry (antioxidants and food dyes) or even drug discovery for various ailments. Evidently, one such plant with these potentials is *B. frutescens*.

The current study showed that *B. frutescens* which is used as a medicinal supplement among some communities possess certain antioxidant qualities. The methanol extract showed significant amount of phenolic content and generally performed well across all the antioxidant assays, especially the *β*-carotene-linoleic acid system assay. The water extract with low phenolic content also displayed strong reducing power and metal chelating ability, and thus suggesting the possibility of other antioxidant substances instead of phenolic compounds. A more in-depth study involving the isolation of the bioactive compounds present is now underway to fully understand the mechanism undertaken by the antioxidants present in the extracts. *B. frutescens* did not exhibit any toxicity against the tested rats, indicating that it may be safe for human consumption. The findings from the current study hold some scientific validation to move forward in the research of *B. frutescens* as a therapeutic agent.
